# Multifocal versus monofocal intraocular lenses after cataract extraction

**DOI:** 10.1590/1516-3180.20151331T2

**Published:** 2014-11-28

**Authors:** Daniel Calladine, Jennifer R. Evans, Sweata Shah, Martin Leyland

## Abstract

**BACKGROUND:**

Good unaided distance visual acuity is now a realistic expectation following cataract surgery and intraocular lens (IOL) implantation. Near vision, however, still requires additional refractive power, usually in the form of reading glasses. Multiple optic (multifocal) IOLs are available which claim to allow good vision at a range of distances. It is unclear whether this benefit outweighs the optical compromises inherent in multifocal IOLs.

**OBJECTIVES:**

The objective of this review was to assess the effects of multifocal IOLs, including effects on visual acuity, subjective visual satisfaction, spectacle dependence, glare and contrast sensitivity, compared to standard monofocal lenses in people undergoing cataract surgery.

**METHODS:**

*Search methods:* We searched CENTRAL (which contains the Cochrane Eyes and Vision Group Trials Register), The Cochrane Library 2012, Issue 2, MEDLINE (January 1946 to March 2012), EMBASE (January 1980 to March 2012), the metaRegister of Controlled Trials (mRCT) (www.controlled-trials.com), ClinicalTrials.gov (www.clinicaltrials.gov) and the WHO International Clinical Trials Registry Platform (ICTRP) (www.who.int/ictrp/search/en). We did not use any date or language restrictions in the electronic searches for trials. The electronic databases were last searched on 6 March 2012. We searched the reference lists of relevant articles and contacted investigators of included studies and manufacturers of multifocal IOLs for information about additional published and unpublished studies.

*Selection criteria:* All randomised controlled trials comparing a multifocal IOL of any type with a monofocal IOL as control were included. Both unilateral and bilateral implantation trials were included.

*Data collection and analysis:* Two authors collected data and assessed trial quality. Where possible, we pooled data from the individual studies using a random-effects model, otherwise we tabulated data.

**MAIN RESULTS:**

Sixteen completed trials (1608 participants) and two ongoing trials were identified. All included trials compared multifocal and monofocal lenses but there was considerable variety in the make and model of lenses implanted. Overall we considered the trials at risk of performance and detection bias because it was difficult to mask patients and outcome assessors. It was also difficult to assess the role of reporting bias. There was moderate quality evidence that similar distance acuity is achieved with both types of lenses (pooled risk ratio, RR for unaided visual acuity worse than 6/6: 0.98, 95% confidence interval, CI 0.91 to 1.05). There was also evidence that people with multifocal lenses had better near vision but methodological and statistical heterogeneity meant that we did not calculate a pooled estimate for effect on near vision. Total freedom from use of glasses was achieved more frequently with multifocal than monofocal IOLs. Adverse subjective visual phenomena, particularly haloes, or rings around lights, were more prevalent and more troublesome in participants with the multifocal IOL and there was evidence of reduced contrast sensitivity with the multifocal lenses.

**AUTHORS’ CONCLUSIONS:**

Multifocal IOLs are effective at improving near vision relative to monofocal IOLs. Whether that improvement outweighs the adverse effects of multifocal IOLs will vary between patients. Motivation to achieve spectacle independence is likely to be the deciding factor.

This is the abstract of a Cochrane Review published in the Cochrane Database of Systematic Reviews (CDSR) 2012, issue 9. Art. No.: CD003169. DOI: 10.1002/14651858.CD003169.pub3 (http://onlinelibrary.wiley.com/doi/10.1002/14651858.CD003169.pub3/pdf/abstract). For full citation and authors’ details see reference 1.
